# Visual properties and perceived restorativeness in green offices: a photographic evaluation of office environments with various degrees of greening

**DOI:** 10.3389/fpsyg.2024.1443540

**Published:** 2024-09-13

**Authors:** Seiji Shibata, Kenro Tokuhiro, Akinori Ikeuchi, Masakazu Ito, Hirotaka Kaji, Masayoshi Muramatsu

**Affiliations:** ^1^Department of Human Psychology, Sagami Women's University, Sagamihara, Japan; ^2^Toyota Central R&D Labs., Inc., Nagakute, Japan; ^3^Toyota Motor Corporation, Toyota, Japan

**Keywords:** green offices, perceived restorativeness, Attention Restoration Theory (ART), Perceptual Fluency Account (PFA), visual properties

## Abstract

Office environments play a critical role in employee wellbeing and productivity. While the benefits of incorporating nature into workspaces have been recognized, the specific visual characteristics that contribute to restorativeness remain unclear. This study investigates how visual characteristics of office environments, specifically the presence of greenery and color complexity, are associated with perceived restorativeness. In Study 1, we developed a scale based on Attention Restoration Theory to measure the restorative characteristics of office environments, consisting of three subscales: Being Away, Fascination, and Extent. In Study 2, we used this scale to examine the correlation between the restorative characteristics of offices and the visual properties of office photographs. The results showed that the square root of the percentage of green area, the color fractal dimension, and the brightness fractal predicted perceived restorativeness. Notably, the color fractal dimension often showed a stronger effect than the amount of greenery per se. These findings suggest that both the presence of greenery and the overall complexity of color transitions in office spaces contribute to their restorative potential. Our study provides insights for designing more restorative office environments, emphasizing the importance of not only increasing greenery but also mimicking natural color patterns. The predictive model developed provides a practical tool for estimating the restorative potential of office designs. Although there are limitations such as the use of photographic assessments and the inability to fully explain the Extent component of restorativeness, this study contributes to our understanding of how to create more psychologically supportive work environments.

## Introduction

While the COVID-19 pandemic increased remote work, physical workplaces remain important for many people. As pandemic restrictions eased, there was a gradual return of workers to office environments. Because of the substantial amount of time spent in these settings, enhancing office environments is vital for improving overall quality of life.

Efforts to improve office environments have varied, with the greening of offices being a notable initiative. Research on office greening has included various perspectives (Bringslimark et al., 2009), including improvements in the thermal characteristics of the office space, such as temperature and humidity (Jumeno and Matsumoto, 2016; Matsumoto, 2009), and air quality (Nishina et al., 1995; Asaumi et al., 1994). Studies have also focused on mental fatigue recovery (Shibata and Suzuki, 2001) and creativity enhancement (Shibata and Suzuki, 2002, 2004). More recently, influenced by the concept of biophilic design advocated by Kellert et al. (2011), there has been a growing interest in the psychological effects of green offices.

###  Restorative effects of exposure to nature

Research on indoor greenery has demonstrated several positive effects on wellbeing and productivity in office environments. Studies have shown that the presence of plants in offices can lead to reduced stress levels (Dijkstra et al., 2008), improved mood (Shibata and Suzuki, 2001), and enhanced cognitive performance (Raanaas et al., 2011). More specifically, Nieuwenhuis et al. (2014) found that enriching office spaces with plants resulted in increased job satisfaction and concentration. In terms of productivity, Knight and Haslam (2010) reported that employees in green offices completed cognitive tasks faster and with fewer errors compared to those in lean offices.

The visual aspects of greenery play a critical role in these benefits. Yin et al. (2010) found that even brief glimpses of green roofs can restore attention and improve performance on cognitive tasks. This suggests that the mere visual presence of greenery can have significant positive effects on cognitive function and wellbeing in office environments.

However, it's important to note that the relationship between the amount of greenery and these benefits is not always linear. Studies by Elbertse and Steenbekkers (2023) and Larsen et al. (1998) suggest that there may be an optimal level of indoor greenery, beyond which additional plants may not provide significant additional benefits. This highlights the need for our study, which aims to investigate the relationship between different levels of office greenery and perceived restorativeness.

Understanding the relationship between office greening and perceived restorativeness is critical for several reasons. First, because employees spend a significant portion of their lives in office environments, optimizing these spaces for wellbeing and productivity is of paramount importance. Second, while the general benefits of exposure to nature are well established, the specific effects of different levels of greenery in office environments remain unclear. This knowledge gap hinders our ability to develop evidence-based guidelines for office design. By focusing on the visual properties of greened office environments, our study aims to provide practical insights that can inform more effective and efficient strategies for creating restorative workspaces. This research is particularly timely given the increasing emphasis on employee wellbeing and the growing trend of incorporating biophilic design elements into office spaces. Our findings have the potential to influence office design practices, potentially leading to improvements in employee satisfaction, productivity, and overall quality of work life.

Numerous studies have shown that natural landscapes have a restorative effect, alleviating mental fatigue and restoring cognitive capacity (Kaplan, 1995; Berto, 2005; Hartig et al., 2003), which is considered one of the psychological benefits of office greening. Two major theories explain the restorative effects of nature exposure: Attention Restoration Theory (ART) (Kaplan, 1995) and Stress Reduction Theory (SRT) (Ulrich et al., 1991). SRT, proposed by Ulrich, focuses on the immediate, unconsciously triggered emotional and physiological responses to nature. It suggests that exposure to natural environments can rapidly reduce stress by evoking positive emotions and decreasing negative feelings. In contrast, ART, developed by Kaplan and Kaplan, emphasizes cognitive processes and the recovery of attentional resources. While both theories acknowledge nature's restorative potential, they differ in their underlying mechanisms and outcomes. SRT primarily addresses affective and physiological restoration, whereas ART focuses on cognitive restoration and the recovery of directed attention.

Our study relies primarily on ART for several reasons. First, ART provides a framework for understanding restorativeness in terms of four key environmental characteristics (Being Away, Fascination, Extent, and Compatibility), which lends itself well to the development of assessment scales. In contrast, SRT's focus on emotional and physiological responses makes it less amenable to the creation of assessment measures based solely on environmental characteristics. Second, many existing restorativeness scales are based on ART (Hartig et al., 1997; Pasini et al., 2014), which allow for better comparability across studies. Finally, ART's cognitive approach aligns well with our focus on visual properties and their potential influence on perceptual fluency, as proposed by the Perceptual Fluency Account (PFA) (Joye and van den Berg, 2011). While we acknowledge the value of SRT in understanding restorative processes, we have determined that basing our research on ART is more appropriate for these reasons, as it allows us to more directly examine how specific environmental characteristics are related to perceived restorativeness in office settings.

Previous research investigating the restorative effects of green offices has predominantly made comparisons based on the presence or absence of plants or on several levels of office greening (Bringslimark et al., 2009). However, these studies faced certain limitations. Comparisons between environments with and without plants, or between environments with many and few plants, involving only a small number of levels, do not allow for a detailed understanding of how changes in the amount of plants are related to the perception of restorativeness. To explore this relationship in detail, it is necessary to study environments with varying amounts of plants. However, systematically changing the amount of greenery in a real office environment has proven challenging, and quantifying the restorative effects of different levels of greenery requires significant sample sizes, making such studies time consuming and expensive.

Research has demonstrated that the restorative effects of nature can be experienced by simply viewing images of natural landscapes (Ulrich et al., 1991; Berto, 2005; Grassini et al., 2019). This finding suggests that photographs can be used to assess the perceived restorativeness of environments, potentially overcoming some of the practical limitations of studies in real office settings. However, while these studies have established the general restorative effects of natural environments, the specific relationship between the amount of greenery in office spaces and perceived restorativeness remains unclear. This knowledge gap limits our understanding of how to optimize the incorporation of greenery into office designs to maximize restorative benefits.

To address this gap, our study aims to investigate how the amount of greenery in an office environment is related to perceptions of restorativeness. By examining a wide range of greenery levels using photographs of offices, we seek to provide a more nuanced understanding of the relationship between office greenery and perceived restorativeness. In addition, by using online surveys, we can efficiently collect data from a large sample size efficiently, addressing another limitation of previous studies.

This research is critical to developing evidence-based guidelines for integrating nature into office spaces to improve employee wellbeing and productivity. By clarifying the relationship between the levels of office greening and perceived restorativeness, we can inform more effective and efficient strategies for creating restorative office environments.

###  Visual properties and restorativeness

Understanding the visual properties of environments and their impact on perceived restorativeness is critical for several reasons. First, it allows us to identify specific elements that contribute to restorative experiences, which can inform evidence-based design practices. Second, it helps bridge the gap between theoretical concepts of restorativeness and practical applications in real-world settings. Finally, by focusing on quantifiable visual properties, we can develop more objective measures of an environment's restorative potential.

Research has shown that restorative effects can be induced by viewing images of natural scenes (Ulrich et al., 1991; Berto, 2005; Grassini et al., 2019). However, it is important to note that the effects of direct and indirect nature exposure may differ in both variety and intensity. While some studies have found similar effects on subjective restoration and affect between real and simulated natural environments (Kjellgren and Buhrkall, 2010), others have reported that direct nature exposure often leads to stronger restorative effects (Browning et al., 2020; Mayer et al., 2009).

In addition, non-visual aspects of nature exposure also contribute to restorative effects. For example, natural sounds have been shown to enhance recovery from stress (Alvarsson et al., 2010), and tactile interactions with natural elements, such as touching plants or feeling natural textures, can also positively influence mood and wellbeing (Koga and Iwasaki, 2013). These findings suggest that while visual properties play a crucial role in the restorative benefits of nature-rich environments, they are not the only contributors.

However, given the significant impact of visual stimuli and the practical advantages of using visual representations in research, our study focuses on the visual aspects of office greening. We acknowledge that this approach may not capture the full range of restorative benefits provided by actual exposure to nature, but it allows us to systematically examine the relationship between visual greenery and perceived restorativeness in office environments. This focus is consistent with ART, which suggests that views of nature are restorative because our information processing mechanisms have evolved to better process natural features that are cognitively less demanding (Kaplan, 1995).

The PFA further emphasizes this point; it posits that the fractal nature of natural scenes promotes perceptual fluency, which, in turn, makes nature restorative and a preferred stimulus (Joye and van den Berg, 2011). Fractals are geometric structures characterized by self-similarity. This property can be observed in various natural phenomena, such as the patterns of tree branches and leaf veins. To illustrate this concept, consider a tree: its overall structure is repeated in its branches, which in turn are repeated in smaller branches and twigs. This self-similarity across different scales makes the tree's structure easier for our visual system to process, despite its apparent complexity. Similarly, the branching patterns of river systems or the intricate details of a fern frond exhibit this fractal-like self-similarity. According to the PFA, our visual system has evolved to efficiently process these natural fractal patterns, resulting in a sense of ease or “fluency” in perception. This perceptual fluency is thought to contribute to the restorative effect of natural scenes. Despite their apparent complexity, these fractal structures exhibit a form of regularity through their self-similar patterns, which reduces the cognitive load of recognition and may contribute to the restorative experience.

In addition to the PFA, numerous attempts have been made to explain preferences for natural landscapes in terms of the visual properties of nature, such as fractality. Previous research has examined relationships between different visual properties, such as color and spatial properties (Kardan et al., 2015; Kuper, 2015), visual-spatial frequencies and power spectra (Valtchanov and Ellard, 2015), and fractality (Hagerhall et al., 2004, 2008, 2015) in the context of landscape preferences.

More recently, Menzel and Reese (2021, 2022) examined the effects of various visual properties of urban and natural landscapes on restorativeness. They hypothesized that natural images would have more properties associated with fluent processing at the early stages in the visual system than urban images. To test this, they used control images that had similar low-level visual properties as the source images (nature and urban), allowing them to examine the influence of these low-level processed properties separately from higher-order processed properties. In their studies, they examined several visual properties, focusing on those most relevant to early visual processing, such as spectral slope and spatial frequency. However, in contrast to the PFA, their results suggested that such low-level properties had little or no direct impact on restoration. This suggests that the restorative effects of exposure to nature likely require higher-level processing, such as object and scene recognition. Nevertheless, their results suggest that lower-level processing may play a mediating or moderating role in the restorative effects of nature images.

Although numerous studies have been conducted with landscapes, research focusing on the visual properties of indoor environments, such as offices, has grown in recent years. Notable contributions include studies of psychological responses to natural patterns in architecture (Coburn et al., 2019), neural responses to architectural interiors (Coburn et al., 2020), aesthetic perception in rapidly presented images including indoor scenes (Mullin et al., 2017), and the effects of fractal light patterns in interiors (Abboushi et al., 2019). Building on this emerging body of research, we sought to explore how specific visual properties are associated with perceived restorativeness in office environments with varying degrees of greenery. Similar to the methods used by Menzel and Reese (2021, 2022), we quantified various visual properties of office photographs, such as color, spatial frequency, and spectral slope, and analyzed their effects on perceived restorativeness. Our study extends previous work by focusing specifically on the interaction between visual properties and greening in office environments, an area that has received less attention in the literature.

The study of visual properties in relation to restorativeness is particularly relevant in the context of office environments. By identifying which specific visual properties contribute most significantly to perceived restorativeness, we can develop more effective strategies for incorporating nature-inspired elements into office designs. Specifically, our research focuses on the fractal properties and fluctuation components present in photographic images of office environments with varying degrees of greenery. By analyzing these characteristics, we aim to understand how they relate to the perceived restorativeness of the space. This knowledge can potentially lead to the creation of work environments that not only look appealing but also actively support cognitive function and wellbeing through their inherent visual structure.

Understanding the visual properties of office environments and their impact on perceived restorativeness is critical to developing evidence-based design practices. Our approach of focusing on quantifiable visual properties allows for more objective measures of an environment's restorative potential. This is particularly relevant in office environments, where employees spend a significant portion of their time. By analyzing specific visual properties such as fractal patterns and fluctuation components, we can bridge the gap between theoretical concepts of restorativeness and practical applications in real-world settings.

Furthermore, this research has implications beyond physical office spaces. Understanding the relationship between visual properties and restorativeness can inform the development of digital interventions or virtual environments that mimic the restorative effects of nature. This could be particularly beneficial in situations where direct exposure to nature is limited, such as in urban environments or during extended periods of indoor work. By identifying the key visual elements that contribute to restorativeness, we can potentially create more effective digital tools for stress reduction and cognitive restoration in various settings.

###  Assessing the restorativeness of environments

In studies of restorative environments, the Perceived Restorativeness Scale (PRS) developed by Hartig et al. (1997) is commonly used to assess the restorative qualities of an environment. This scale relies on ART to measure the perceived restorativeness of an environment. According to ART, four primary elements make up a restorative environment: Being Away, Fascination, Extent, and Compatibility. Being Away includes attributes that promote a sense of psychological and spatial separation from daily stressors. Fascination refers to attributes that effortlessly capture one's attention. Extent includes attributes that evoke a sense of spatial and temporal expansiveness. Compatibility refers to the congruence between the behavioral demands of a given setting and the environment's provisions.

The PRS was originally developed to assess outdoor spaces and therefore includes items that are not suitable for evaluating indoor spaces, such as offices. In addition, the scale has more than 20 items, making it impractical to use it repeatedly to evaluate a large number of photographs. Therefore, we first developed a scale similar to PRS and based on ART to evaluate indoor environments, and then applied the scale to an office setting.

Although ART consists of four components that contribute to an environment's restorativeness, we did not include Compatibility in our scale. Compatibility refers to the congruence between the behavioral demands of a given setting and the environment's provisions. While we acknowledge the importance of Compatibility, our study focused primarily on the visual properties of environments that can be assessed through photographs. Compatibility is highly context dependent and involves complex interactions between individual goals, tasks, and environmental features that go beyond visual properties alone.

Accurately measuring Compatibility would require detailed information about each participants' work demands and long-term observations in actual office environments, which was beyond the scope of our image-based study. However, we recognize that future research examining the relationship between greening in office environments and Compatibility would be valuable. This could include field studies in real office environments or more comprehensive research designs that take into account specific work demands.

Our decision to exclude Compatibility is also supported by previous studies that have reported that the Compatibility item does not consistently form a single factor within the PRS (Hartig et al., 1997; Pals et al., 2009; Pasini et al., 2014). Nevertheless, we believe that our focus on visual properties provides important insights into how office greenery is related to perceived restorativeness, which can serve as a basis for more comprehensive evaluations of office environments in future research.

###  Objectives of the studies

This study aimed to clarify the relationship between the restorativeness of green offices and the visual properties of the office, such as the amount of green, color, and fractal structures. We conducted two studies. In Study 1, we developed a concise and effective scale based on ART, to measure the restorative properties of the office. In Study 2, we evaluated the restorative properties of the office using the scale developed in Study 1 and analyzed the correlation between the restorative scale score and the visual properties of the office photographs.

By quantifying various visual properties of office photographs and analyzing their impact on perceived restorativeness, we aim to provide designers and facility managers with concrete, actionable insights. This approach can potentially lead to more precise guidelines for creating restorative office environments, moving beyond general recommendations to incorporate greenery toward specific, evidence-based design strategies.

Based on the theoretical framework and previous findings, we formulated several hypotheses for this study. We predicted that perceived restorativeness would increase with the amount of greenery in office environments, based on ART's assertion that the amount of greenery leads to greater perceived restorativeness (Kaplan, 1995). In addition, we hypothesized that certain visual properties associated with natural elements would be positively correlated with perceived restorativeness. This prediction is based on the PFA, which suggests that certain visual properties of natural scenes, such as fractality, contribute to ease of processing and, consequently, to restorativeness (Joye and van den Berg, 2011). Furthermore, we expected that the relationship between greenery and restorativeness would be partially mediated by these visual properties. This hypothesis stems from recent research suggesting that while low-level visual properties may not be directly associated with restoration, they may play a mediating role in the restorative effects of natural images (Menzel and Reese, 2021, 2022). These hypotheses guided our research design and analysis, allowing us to systematically examine the complex relationships among office greening, visual properties, and perceived restorativeness.

###  Ethical statement

The procedure of this study was in accordance with the Declaration of Helsinki and was approved in advance by the Sagami Women's University Ethical Review Committee for Research involving Human Participants/Sagami Women's Junior College Ethical Review Committee for Research involving Human Participants (Acceptance No. 21021), the Research Ethics Review Committee of Toyota Motor Corporation (Approval No. 2022TMC0001), and the Experimental Ethics Review Committee of Toyota Central R&D Labs., Inc. (Reference No. 21-05). Informed consent was obtained from the participants at the survey site, including details about the purpose of the survey and the privacy policy.

## Study 1

In Study 1, we selected scale items that effectively measure the restorative properties of an office, based on ART. We focused on minimizing the number of items selected to facilitate the use of the scale in future studies that require repeated assessments of different environments while maintaining high accuracy.

###  Methods

#### Participants

Participants were recruited through Yahoo Crowd Sourcing, and the initial sample consisted of 1,241 individuals (613 males and 628 females, aged 30–59 years). Participants who completed the entire survey received points through Yahoo. These points could be used to pay for online purchases and served as an incentive for participation. We included two attention check questions throughout the survey. These checks consisted of instructional manipulation checks (e.g., “Please select ‘Strongly Agree' for this question”). Participants who failed at least one of these attention checks were excluded from the final analysis. After applying this exclusion criterion, the final sample size consisted of 1,167 individuals (575 males and 592 females, aged 30–59 years).

#### Materials

**Figure 1:**
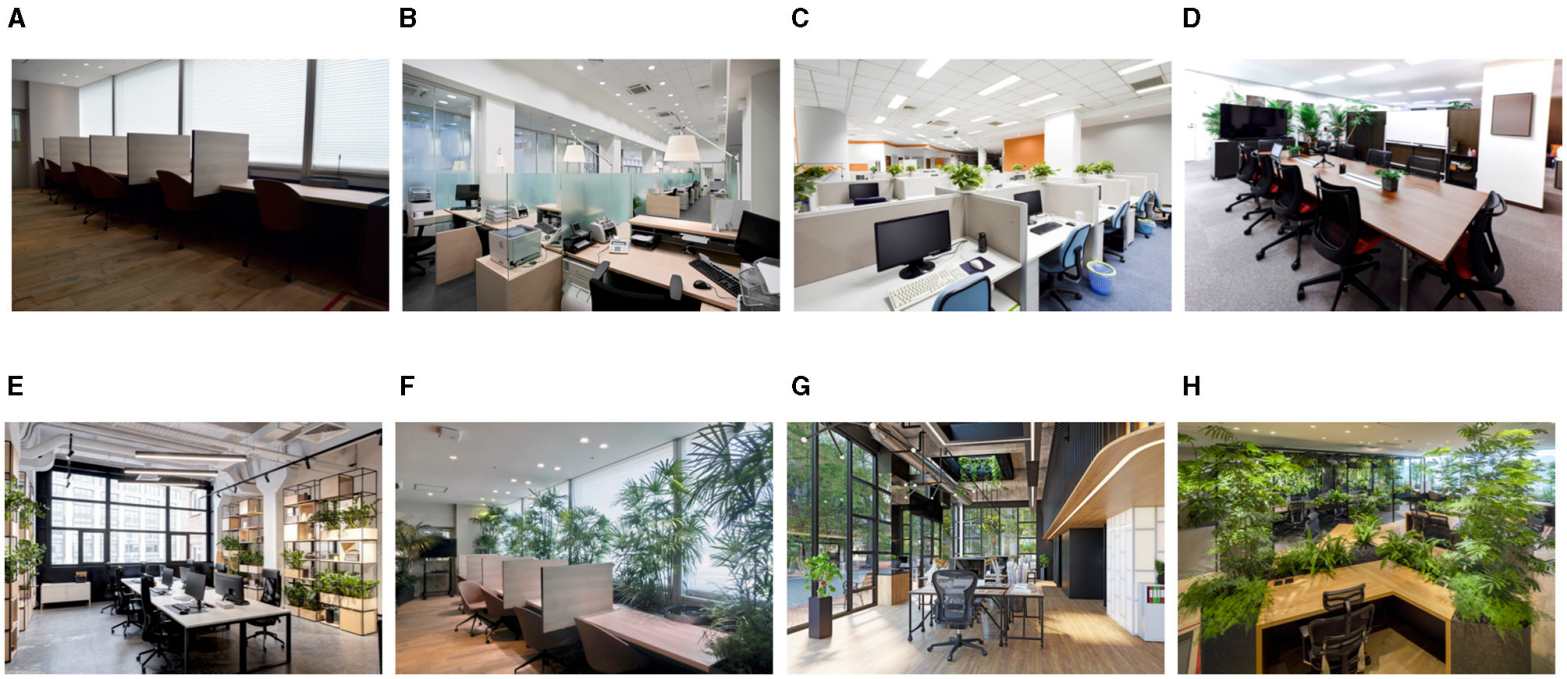
Photographs of offices used in the survey. **(A)** Office 1. **(B)** Office 2. **(C)** Office 3. **(D)** Office 4. **(E)** Office 5. **(F)** Office 6. **(G)** Office 7. **(H)** Office 8.

We used eight office photographs (Figure 1), three of which were taken in at the Genki-Office^Ⓡ^ (offices 1, 6, and 8) located within the Toyota Frontier Research Center. The remaining five photographs were taken from various online materials.

For Study 1, which aimed to develop a scale for assessing the restorativeness of indoor environments, we selected a variety of office photographs to ensure variability in restorativeness ratings. Due to the limited availability of images of green offices, we used a combination of publicly available photographs and images taken in the Genki-Office. While these images vary in aspects beyond just the amount of greenery, this diversity was necessary to create a robust scale applicable to different office environments.

The photographs were taken from an angle that provides a comprehensive view of the office space, including the surroundings of typical workstations. Although this perspective differs from the direct line of sight of a seated worker, it captures the broader visual context that employees experience throughout their workday, including as they move around the office or shift their gaze. This approach allows for a more holistic assessment of the restorative potential of the office environment.

#### Rating scale

The scale to assess the restorative properties of office environments (Indoor Restorative Characteristic Scale: IRCS) was developed based on ART (Kaplan, 1995). The preliminary version of the scale was developed through a collaborative process involving all authors of the current study. We began by carefully reviewing the definitions of Being Away, Fascination, and Extent within ART. Based on these definitions, we generated new items and adapted relevant items from existing scales, such as the PRS (Hartig et al., 1997). This process resulted in 11 items for Being Away, 10 for Fascination, and 9 for Extent. Approximately 70% of the items were newly created, while the remaining 30% were adapted from existing scales, with modifications to fit the context of indoor office environments. Each item on the scale was rated on a five-point scale, ranging from “not at all” (1) to “very much” (5). The survey was conducted in Japanese, as all participants were native Japanese speakers. For the purpose of international dissemination, English translations of the items are provided in Supplementary material A.

In addition, we used the five-item restorative experience scale (Shibata, 2022) to assess the restorativeness of each photograph and to test the concurrent validity of our newly developed scale. The translated items of the scale are provided in Table 1. Each item was scored on a five-point scale, ranging from “not at all” (1) to “very much” (5). The restorativeness score was calculated by averaging the scores of the five items.

**Table 1:** Items in the scale of the restorative experience.

**Item**
I experience a sense of lightness in my heart
My mind feels clear and focused
I experience a refreshed and invigorated mood
My fatigue dissipates
I feel as though my mind has been cleansed

All evaluation items used in the survey were in Japanese. Here, their English translations are provided.

#### Procedure

The survey was conducted online using LimeSurvey (Limesurvey GmbH, 2023) to create a dedicated survey website. Ethical approval was obtained at each of the authors' institutions before data collection began. Once participants were informed of the purpose of the survey, they provided consent on the participant recruitment page of Yahoo Crowd Sourcing and were directed to the survey site.

After providing their age and gender, participants were presented with one of eight office photographs with a size of 480 (width) × 324 (height) pixels, accompanied by the instruction, “Imagine you are in the room shown in this picture. To what extent do you experience the impressions listed below in this room?” Participants were then asked to rate each item displayed below the photograph. For the IRCS ratings, six items were shown at a time. After rating the six items, participants were presented with another set of six items, and this process was repeated until all 30 items had been rated. The order of the items was randomized for each participant. After rating all IRCS items, participants were shown the same photograph on the subsequent page and asked to rate the photograph based on the five items of the restorative experience scale with the instruction: “If you were to spend some time in this room, to what extent would you experience the feelings described in the following statements?”

#### Statistical analysis

Items with sufficiently high loadings on factors corresponding to each component of the restorative properties in ART were first selected using exploratory factor analysis. To keep the number of items to a minimum, we then used bi-factor analysis to select items that were consistent with the overall scale but provided information specific to each subfactor. The psych (Revelle, 2023) and lavaan (Rosseel, 2012) packages in R (R Core Team, 2023) were used for the analysis. After selecting the rating scale items, we confirmed the convergent validity of the developed scale by correlating the score of the selected items with the score of the restorative experience scale.

###  Results

For the initial analysis of the IRCS ratings, we conducted parallel analysis to determine the number of factors. The results of the parallel analysis indicated the presence of six underlying factors. Subsequent maximum likelihood estimation of factor analysis with oblimin rotation revealed that Factor 1 had high loadings on the Being Away items, Factor 2 on Fascination, and Factor 3 on Extent. Additionally, Factor 4 had high loadings on items describing the absence of distraction, while Factor 5 was related to the coherence of the environment. Factor 6 had high loadings on only two items and accounted for a small proportion of the explained variance. In light of these results, we decided to retain a total of 15 items with absolute loadings of 0.4 or higher for Factors 1–3 (seven items for Being Away, five items for Fascination, and three items for Extent), which represent the basic components of ART.

**Figure 2:**
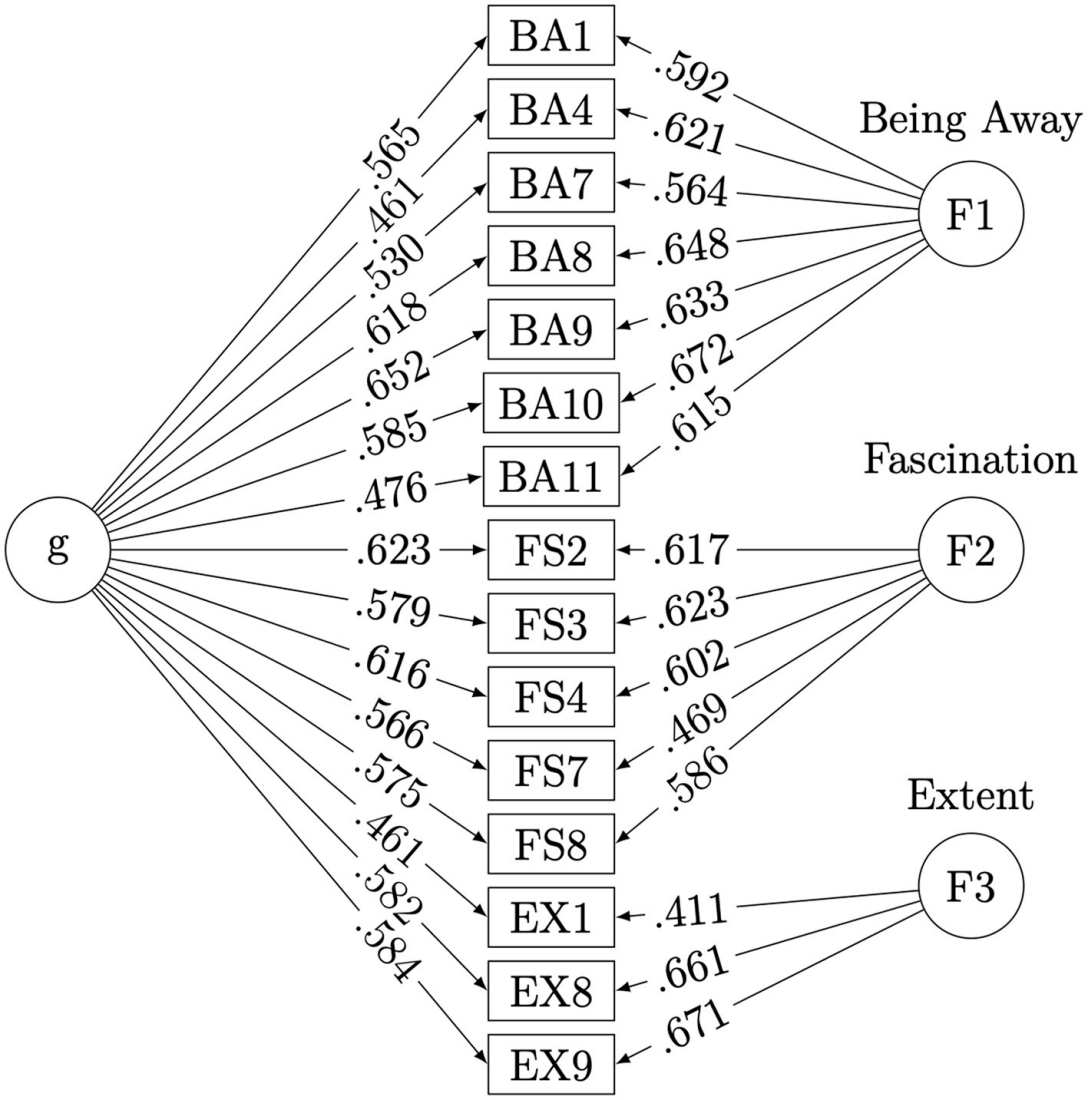
Resulting path diagram from the bifactor analysis.

We then used the bi-factor analysis for further item selection. The resulting path diagram of the bi-factor analysis using the 15 items is shown in Figure 2. In the bi-factor analysis, we focused on items that had high loadings on both the general and group factors. In addition, because there were three items for Extent, four items were selected for both Being Away and Fascination to ensure that each factor had a similar number of items, and that the resolution of the measurement was not too low.

For Being Away, four items (BA1, BA7, BA9, and BA10) were selected based on their face validity from the five items with loadings of 0.5 or higher on both the general and group factors. For Fascination, four items (FS2, FS3, FS4, and FS8) with loadings of 0.5 or higher on both the general and group factors were selected. The scale for rating the restorative properties of offices contained a total of 11 items (four items each for Being Away and Fascination and three items for Extent). The McDonald's omegas for the scale were ω_*t*_ = 0.946 and ω_*h*_ = 0.686, indicating adequate internal consistency.

We calculated the mean scores of each subscale and then calculated the overall Restorativeness score as the mean of these three subscale scores. The means and standard deviations of each score and Pearson's correlations between the scores are shown in Table 2.

**Table 2:** Means, standard deviations (SD), and correlation matrix of the restorative experience scale and restorative characteristic scores.

	**1**	**2**	**3**	**4**	***M***	***SD***
1. Restorative experience	–				2.72	1.02
2. Overall restorativeness	0.83^***^	–			2.80	0.79
3. Being away	0.82^***^	0.80^***^	–		2.64	1.05
4. Fascination	0.57^***^	0.79^***^	0.46^***^	–	2.62	0.95
5. Extent	0.57^***^	0.79^***^	0.43^***^	0.45^***^	3.15	1.00

^***^p < 0.001.

As shown in Table 2, there was a high correlation between the Restorative Experience score and the Overall Restorativeness score (*r* = 0.83). The Being Away score had the highest correlation with the Restorative Experience score (*r* = 0.82), and the Fascination and Extent scores had moderate positive correlations with the Restorative Experience score (*r* = 0.57 for both). Among the restorative characteristic subscale scores, the Being Away, Fascination, and Extent subscale scores all showed a strong positive correlation with the Overall Restorativeness score (*r* = 0.80, 0.79, and 0.79, respectively). The correlations between these subscales ranged from 0.43 to 0.46.

###  Discussion

In Study 1, we developed the IRCS, a scale based on ART to assess the restorative properties of an office environment. We developed an 11-item scale consisting of three subscales: Being Away, Fascination, and Extent. The high positive correlation between the overall score of the developed scale and the restorative experience score indicated that the convergent validity of this scale was adequate. In addition, although the correlation between the overall score and each of the three subscale scores was high, the correlations among the subscales were of moderate strength, indicating that the discriminant validity of the three subscales was also adequate.

## Study 2

When evaluating the perceived restorativeness of an environment using photographs, it is generally assumed that such an assessment is based on certain inherent visual properties of the photographs. However, studies that have specifically examined the relationship between these visual properties and perceived restorativeness are limited. Therefore, in Study 2, we sought to explore this relationship using the scale we developed in Study 1, with a focus on the perceived restorativeness of an office environment and the visual properties of office photographs.

This study sought to clarify the relationship between the restorativeness of green offices and specific visual properties, such as the amount of green, color characteristics, and fractal structures. By quantifying various visual properties of office photographs and analyzing their impact on perceived restorativeness, we aimed to provide designers and facility managers with concrete, actionable insights. This approach can potentially lead to more precise guidelines for creating restorative office environments, moving beyond general recommendations for incorporating greenery to specific, evidence-based design strategies.

Based on the theoretical framework and previous findings, we formulated several hypotheses for this study. We predicted that perceived restorativeness would increase with the amount of greenery in office environments, based on ART's assertion that the amount of greenery leads to greater perceived restorativeness (Kaplan, 1995). In addition, we hypothesized that specific visual properties associated with natural elements would be positively correlated with perceived restorativeness. This prediction is grounded in the PFA, which suggests that certain visual properties of natural scenes, such as fractality, contribute to ease of processing and, consequently, to restorativeness (Joye and van den Berg, 2011). These hypotheses guided our research design and analysis, allowing us to systematically investigate the relationships between office greenery, visual properties, and perceived restorativeness.

###  Methods

#### Participants

Ethical approval was obtained at each of the authors' institutions before we began data collection. In Study 2, we collected data from 1,500 participants between the ages of 30 and 59. The participants were recruited through Yahoo Crowd Sourcing. As in Study 1, participants who completed the entire survey were awarded points by Yahoo. We implemented attention checks to ensure data quality. For each of the five office images evaluated, we included one attention check item (e.g., “Please select ‘Strongly Agree' for this question”). Participants who failed to respond correctly to any of these attention checks across the five image ratings were excluded from the analysis. After excluding participants who faild the attention checks, the final sample size consisted of 1,322 individuals (659 males and 663 females).

The sample size for this study was determined based on practical considerations and the goal of obtaining a sample that would be considered more than adequate by conventional standards in the field. We aimed to collect a large sample to ensure robust results, given the exploratory nature of some aspects of our research.

Our study design included 60 office photographs, divided into 12 groups of five photographs each. Each group was rated by ~100 participants. For our main analyses, we used the mean rating scores for each photograph.

#### Materials

We used 60 photographs of offices with varying degrees of greening (see Supplementary material B for the list of the photographs). To ensure consistency in image quality and characteristics for our visual analysis, all photographs were taken by one of the authors using the same camera settings and composition guidelines. The photographs were taken at several cooperating offices in Japan, including shared offices, single-person offices, and the Genki-Office^Ⓡ^. We included photographs from the Genki-Office due to the limited number of accessible green offices and the need for a wide range of greenery levels in our study.

All photographs were taken from an angle that provided a comprehensive view of the area around the seat, using a wide-angle lens (focal length 24 mm). This approach allowed us to capture a broader context of the office environment, which is important for assessing perceived restorativeness. To standardize the images and focus on the structural and design elements of the offices, we removed personal items and ensured that no people were present in the photographs. We acknowledge that this may not fully represent a “lived-in” office space, but it allowed for more consistent analysis of visual properties.

The photographs included a variety of modern office designs, ranging from traditional layouts to more contemporary open-plan and flexible workspaces. This diversity reflects current trends in office design and allows for a more comprehensive assessment of restorativeness across different office types.

**Figure 3:**
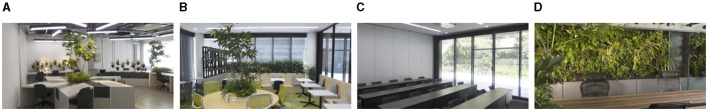
Samples of the office photographs used in Study 2. **(A)** Photo 1. **(B)** Photo 2. **(C)** Photo 3. **(D)** Photo 4.

Figure 3 shows sample photographs used in the study. Each photograph (4,808 pixels in width and 3,456 pixels in height) was cropped to 4,096 pixels × 2,048 pixels from the center, and further resized to 640 (width) × 320 (height) pixels for display on the survey page. This standardization process ensured that all images had consistent resolution and aspect ratio for our visual property analysis.

To minimize the burden of rating a large number of photographs while ensuring that a wide range of greenery was represented, we divided the 60 photographs into 12 groups of five photographs each. The grouping was based on the degree of greenery in each photo. The photos were classified based on the calculated greenery ratio and divided into 12 groups so that the degree of greenness was relatively consistent among the groups. This approach resulted in each group containing a range of greenery levels, from low to high, while maintaining a similar average level of greenery across groups.

Each participant was randomly assigned to one of the 12 groups and presented with the five photographs from that group. This design means that greenery level was a within-subject factor for each participant, as they saw a range of greenery levels, but the specific set of photographs varied between participants.

We acknowledge that this approach means that each participant saw only a subset of the full range of greenery levels. However, by having a large number of participants and randomly assigning them to different groups, we were able to collect data across the full range of greenery levels while keeping the task manageable for individual participants. The final sample size for each group after excluding participants who failed the attention check, is shown in Table 3.

**Table 3:** Final sample size for each group.

	**Group**											
	**1**	**2**	**3**	**4**	**5**	**6**	**7**	**8**	**9**	**10**	**11**	**12**
Male	51	64	54	37	54	62	51	61	64	56	56	53
Female	51	63	45	51	58	59	58	76	43	42	59	54
Total	102	127	99	88	112	121	109	137	107	98	115	107

#### Measures

##### Restorative characteristics

The restorative characteristic of each office was assessed using the IRCS we developed in Study 1. Each item was rated on a five-point scale, ranging from 1 (not at all) to 5 (very much), and the mean of the response scores was used to calculate each subscale score. The reverse items in the Extent subscale were inverted before the mean score was calculated. The mean of the three subscale scores was calculated and used as the overall Restorativeness score. We combined participants' ratings for each photograph and then averaged the ratings to calculate the restorative characteristic score for each photograph.

##### Visual properties

Of all the visual properties of the photographs, the amount of greenery was first calculated as the most basic attribute. To quantify this, we used Python 3.9.12 with the OpenCV-Python 4.6.0 module. The color image data, consisting of 8-bit red, green, and blue channels, was converted to 8-bit hue, saturation, and value (HSV) channels. Pixels were counted as “green” if they met the following criteria: Hue between 50 and 170, Saturation between 20 and 100, and Value between 10 and 100. The amount of greenery was then calculated as the percentage of green pixels to the total number of pixels in the image (i.e., green area/photo area × 100). The amount of greenery in the photographs ranged from 0.00 to 60.18%.

It is important to note that this method does not distinguish between natural and artificial green elements in the images. While it provides an objective measure of the overall “greenness” of each photograph, it may include non-plant green elements such as green furniture or decorations. This limitation should be considered when interpreting the results of our analyses relating greenery to perceived restorativeness.

We also calculated fractal and fluctuation indices of shape and color (hue, brightness, and saturation) as additional visual properties. Fractal indices measure patterns of self-similarity in the image across scales that are often found in natural elements and can contribute to visual complexity and interest. These include geometric fractals (e.g., the repeating patterns in edge structures) and statistical fractals (e.g., the distribution of intensity across different scales).

Shape Fractal measures the complexity and self-similarity of the image's edges and shapes. Higher values of Shape Fractal indicate more complex and intricate edge patterns, while lower values indicate simpler, more uniform shapes. Geometric Fractal Dimension quantifies the spatial complexity of the image, with higher values indicating more complex and detailed spatial structures at various scales. The Statistical Fractal Dimension measures the roughness and irregularity of the image, with higher values indicating more irregular and rough textures at different scales.

For both the geometric and statistical fractal dimensions, we computed separately for smaller and larger scales, as well as for overall fractal dimensions. This approach allows for a multi-scale analysis of the image, capturing both fine local features and global structures. The Color Fractal Dimension quantifies the complexity of the color distribution in the image, with higher values indicating more complex and varied color distributions.

Fluctuation indices, such as 1/f fluctuation, quantify the variability and rhythmic patterns in the brightness and saturation of the image. 1/f fluctuation measures the balance between randomness and order in the image, with values significantly different from 1 indicating either too much randomness or too much order.

Both fractal and fluctuation measures are often associated with natural scenes (e.g. Joye, 2007; Patuano, 2018; Coburn et al., 2019; Isherwood et al., 2021) and may be associated with visual preference and perceived restorativeness. In total, we analyzed 19 visual properties, including the square root of the amount of greenery, mean brightness and saturation, several fractal dimensions, and fluctuation indices. These indices provide quantitative measures of visual complexity and variability that may be associated with perceived restorativeness. See Supplementary material C for the full list of indicators used in the analysis and Supplementary material D for the definitions of each indicator.

#### Procedure

The survey was created using LimeSurvey and administered online. Participants were informed of the purpose of the survey and provided consent on the participant recruitment page of Yahoo Crowd Sourcing before being directed to the survey site. They were then randomly assigned to one of 12 groups.

After providing their age and gender, participants were presented with one of the five assigned office photographs. The order of the photos was randomized for each participant. Participants were then asked to rate the photo on the IRCS items displayed below the photo. The order of the 11 items was randomized to ensure that each subscale was not grouped together, while remaining consistent across photos and participants. Once all items were rated, the participants were then presented with the next photograph and were asked to rate it using the same scale. This procedure was repeated until all five photographs assigned to the participant had been rated.

#### Statistical analysis

We obtained the mean ratings of the restorative characteristics for each photograph and analyzed their relationship to the visual properties of the photographs. The analysis consisted of two parts. First, we performed a correlation analysis to examine the relationship between the amount of greenery in the office photographs and the scores of overall restorativeness and its subcomponents. Second, we examined how different visual properties, including the amount of greenery, were associated with perceptions of restorativeness in the office.

To address potential effects of photograph grouping on our analysis, we conducted additional analyses using mixed-effects models with the photograph group as a random effect. However, the variance of the random effect was found to be negligible (i.e., σ^2^ < 0.001 in every model), and model comparison based on the Akaike Information Criterion (AIC) indicated that the model without the random effect provided a better fit to the data (consistently ΔAIC = 2 in every model). Given these results, we proceeded with our original approach that did not account for photograph grouping in our final analysis.

To analyze the effect of different visual properties on perceived restorativeness, we used lasso regression (Tibshirani, 1996), a method commonly used in machine learning, to distinguish between variables that are more strongly associated with perceived restorativeness and those that are not, and to obtain a more parsimonious explanatory model. Lasso regression is a technique that adds a constraint called “regularization” to standard multiple regression analysis, setting the coefficients of less influential variables to zero and selecting only important variables. This feature allows lasso regression to perform variable selection and prevent overfitting at the same time. As a result, it can produce a more parsimonious and interpretable model, with the potential to improve predictive accuracy.

Prior to the lasso regression, we addressed multicollinearity issues among the 19 indicators computed as image properties. We first calculated the variance inflation factor (VIF) for all indicators. We then iteratively removed the variable with the highest VIF and repeated this variable selection process. In this process, the square root of the amount of greenery was excluded from potential elimination due to its importance in the study. This process was repeated until all of the variables that remained had a VIF of < 5. As a result, nine variables were retained: the square root of the amount of greenery, mean brightness, mean saturation, shape fractal, large scale geometric fractal of brightness, large scale statistical fractals of brightness and saturation, color fractal, and 1/f fluctuation of brightness. The Pearson's correlation matrix of the nine variables finally selected is shown in Table 4.

**Table 4:** Pearson's correlation matrix of nine variables to be entered as explanatory variables in lasso regression.

	**1**	**2**	**3**	**4**	**5**	**6**	**7**	**8**
1. Square root of greenery	–							
2. Mean brightness	–0.45^*^	–						
3. Mean saturation	0.71^*^	–0.70^*^	–					
4. Shape fractal	–0.21	0.35^*^	–0.19	–				
5. Geometric fractal of brightness	0.42^*^	0.00	0.07	–0.14	–			
6. Statistical fractal of brightness	0.43^*^	–0.14	0.26^*^	–0.07	0.51^*^	–		
7. Statistical fractal of saturation	0.28^*^	0.16	0.01	0.03	0.38^*^	0.42^*^	–	
8. Color fractal	–0.39^*^	–0.15	–0.11	0.02	–0.36^*^	–0.50*	–0.29^*^	–
9.1/f Fluctuation of brightness	0.16	0.34^*^	–0.34^*^	–0.31^*^	0.59^*^	0.14	0.28^*^	–0.35*

^*^p < 0.05.

To account for the potential effect of variable range on variable selection in lasso regression, we standardized all predictor variables prior to analysis. In implementing the lasso regression, we used 10 out of the 60 images as test data, and the remaining 50 as training data. The extraction of test data was done randomly (the random seed value was fixed to maintain reproducibility of the results). To calculate the optimal value of lambda, the parameter that determines the strength of regularization in lasso regression, we performed 10-fold cross-validation using the 50 training data. This method involves dividing the training data into 10 parts, using nine subsets for training and the remaining one for validation. This process is repeated 10 times, ensuring that each subset is used once as validation data. This approach allows for more accurate evaluation of model performance and prevents overfitting. It also maximizes the use of limited data sets by using all data points for both training and validation.

In determining the optimal value of lambda, we used the one standard error rule (Hastie et al., 2009). This method determines the value of lambda to obtain the most parsimonious model within the range of one standard error of the minimum root mean square error (RMSE) of the regression model. Using this approach, we performed variable selection on the training data, then applied the resulting model to the test data to confirm that it could adequately explain the data not used in training (i.e., that overfitting to the training data had not occurred).

The glmnet package (Friedman et al., 2010) in R was used for the lasso regression analyses.

###  Results

#### Amount of green and perceived restorativeness

The Overall Restorativeness scores yielded standard deviations < 1 for 59 out of the 60 photographs. The standard deviation for the remaining photograph was 1.01, indicating consensus in the ratings among participants. The number of participants rating each image varied and is detailed in Supplementary material B.

**Figure 4:**
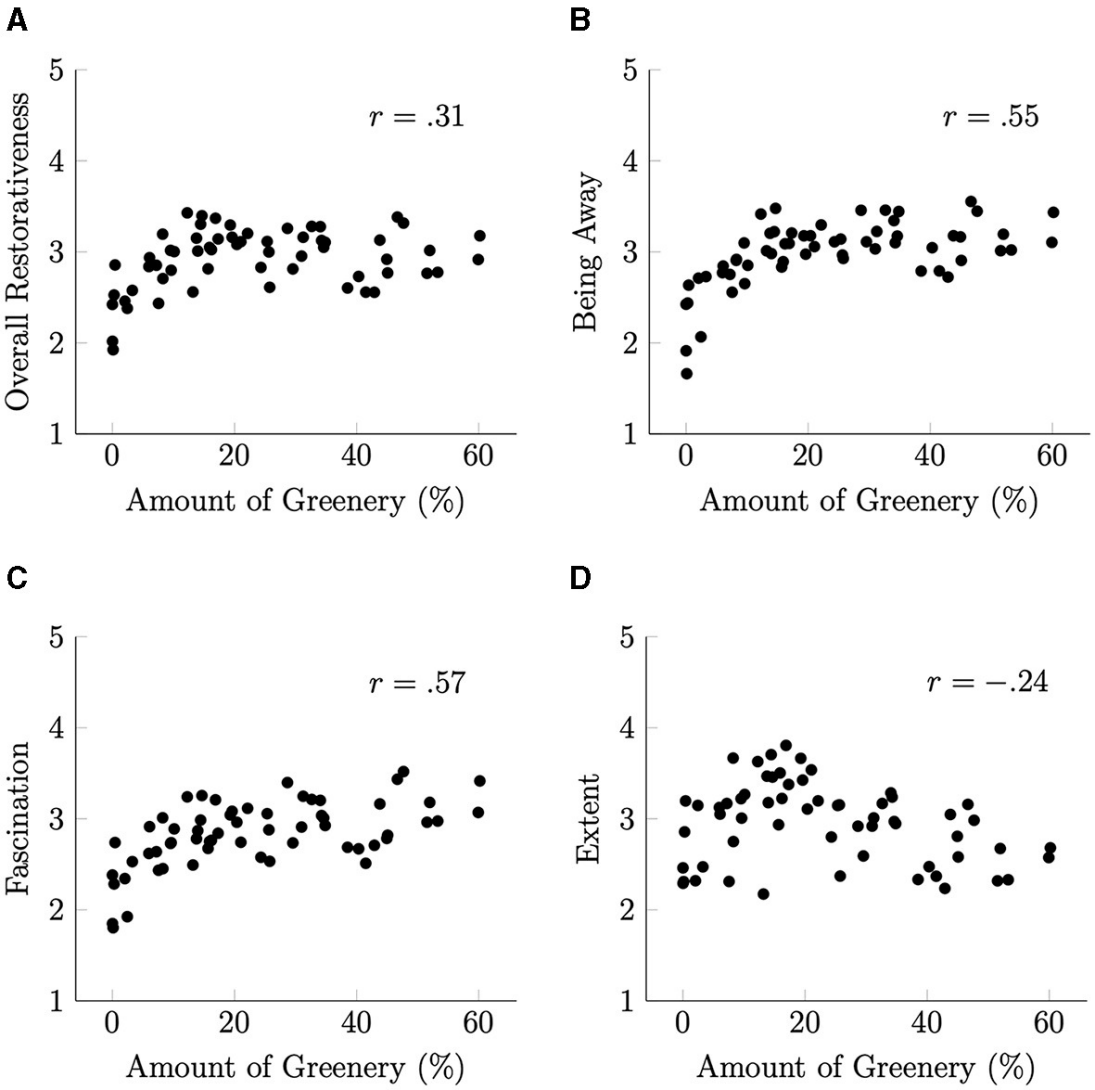
Scatterplots illustrating the relationship between the amount of greenery and the restorative characteristic scores. **(A)** Overall restorativeness. **(B)** Being away. **(C)** Fascination. **(D)** Extent.

As shown in Figure 4, the relationships between the amount of greenery and the Overall Restorativeness, Being Away, and Fascination scores appear curvilinear. The scores increase significantly when the amount of greenery is very low, but the change in score becomes smaller as the amount of greenery moves from a moderate to a high level. To address this non-linear relationship, we explored alternative representations of the data.

Because the amount of greenery calculated for each photograph was represented by a percentage of the green area relative to the total area, we converted these values to the square roots of the percentages. This transformation is supported by research on the density perception. Anobile et al. (2015) claimed that Weber's law operates when people judge very coarse texture densities and that the square root law operates at densities above a certain level. Our results may be explained by this difference in density judgments.

**Figure 5:**
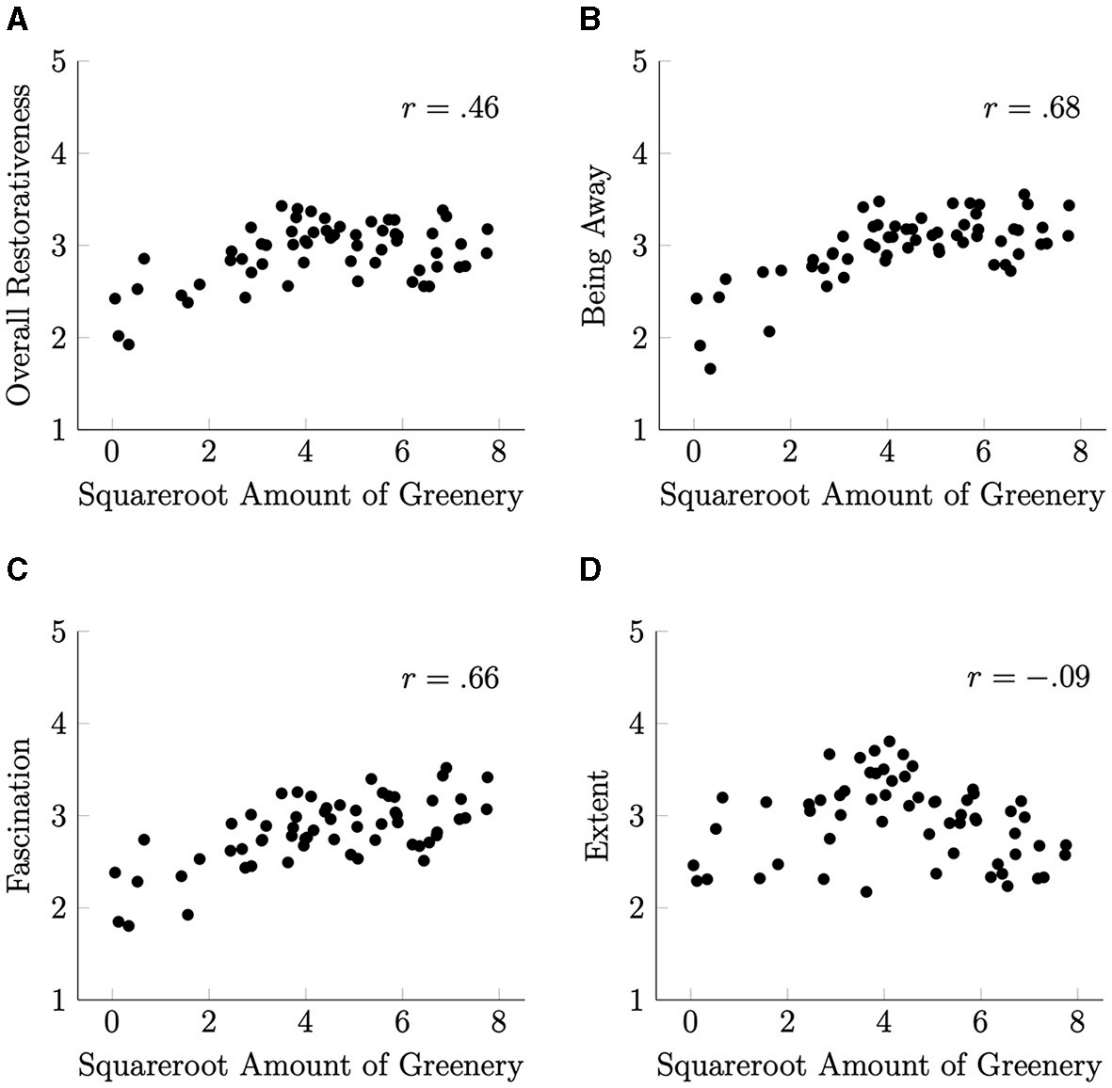
Scatterplots illustrating the relationship between the square root of the amount of greenery and the restorative characteristic scores. **(A)** Overall restorativeness. **(B)** Being away. **(C)** Fascination. **(D)** Extent.

Figure 5 shows the scatterplots illustrating the relationship between the square root of the amount of greenery and the restorative property scores. This transformation allowed for a clearer visualization of the relationships, and Pearson's correlation coefficients increased.

Of the Restorativeness subscale scores, those for Being Away and Fascination were strongly correlated with the square root of the amount of greenery (*r* = 0.68 and .66, respectively). In addition, the Overall Restorativeness score showed a moderate correlation with the square root of the amount of greenery (*r* = 0.46). The score for Extent was not significantly correlated with the square root of the amount of greenery (*r* = −0.09).

#### Analysis including additional visual properties

The predictor variables selected by lasso regression using the lambda obtained by cross-validation are shown in Table 5. As shown in Table 5, there is no large discrepancy between the RMSE for the training data and the RMSE for the test data in any of the models, indicating that the models do not overfit to the training data.

**Table 5:** Obtained models through cross-validated lasso regression.

	**Overall restorativeness**	**Being away**	**Fascination**	**Extent**
Intercept	2.910	2.972	2.822	2.928
Square root of greenery rate	0.010	0.114	0.099	
Geometric fractal of brightness	0.035	0.005	0.005	
Statistical fractal of brightness	0.033		0.062	
Color fractal	–0.121	–0.106	–0.101	
RMSE (trained data)	0.231	0.247	0.216	0.437
RMSE (test data)	0.256	0.241	0.245	0.420

The values shown here are the coefficients of the lasso model created using the training data. All predictors are standardized in the analyses. Thus, all the coefficients except the intercepts are standardized. The estimation process of lasso regression is non-linear, and the standard error calculation method used in conventional linear regression cannot be applied. Therefore, the standard errors of the coefficients are not calculated, and significance tests for individual coefficients are not performed. As indicators of model fit, we present the RMSE for the training data and the RMSE for the test data.

For the models of Overall Restorativeness, Being Away, and Fascination, the square root of greenery percentage and color fractal were commonly selected as valid predictor variables. In addition, the geometric fractal of brightness was selected for all three models, while statistical fractal of brightness was selected for Overall Restorativeness and Fascination. No predictor variables were chosen for Extent.

Based on the standardized coefficients, the color fractal appears to be the strongest predictor for Overall Restorativeness, Being Away, and Fascination, consistently showing the largest negative effect. The square root of the greenery percentage shows the second strongest effect for Being Away and Fascination, with a positive correlation. For Overall Restorativeness, while the square root of the greenery percentage was a valid predictor, but its effect was smaller than both the geometric and statistical fractals of brightness.

These results suggest that the color complexity (represented by color fractals) and the amount of greenery are the most influential factors in perceived restorativeness, while brightness patterns (geometric and statistical fractals) play a secondary but still significant role in some aspects of restorativeness.

**Figure 6:**
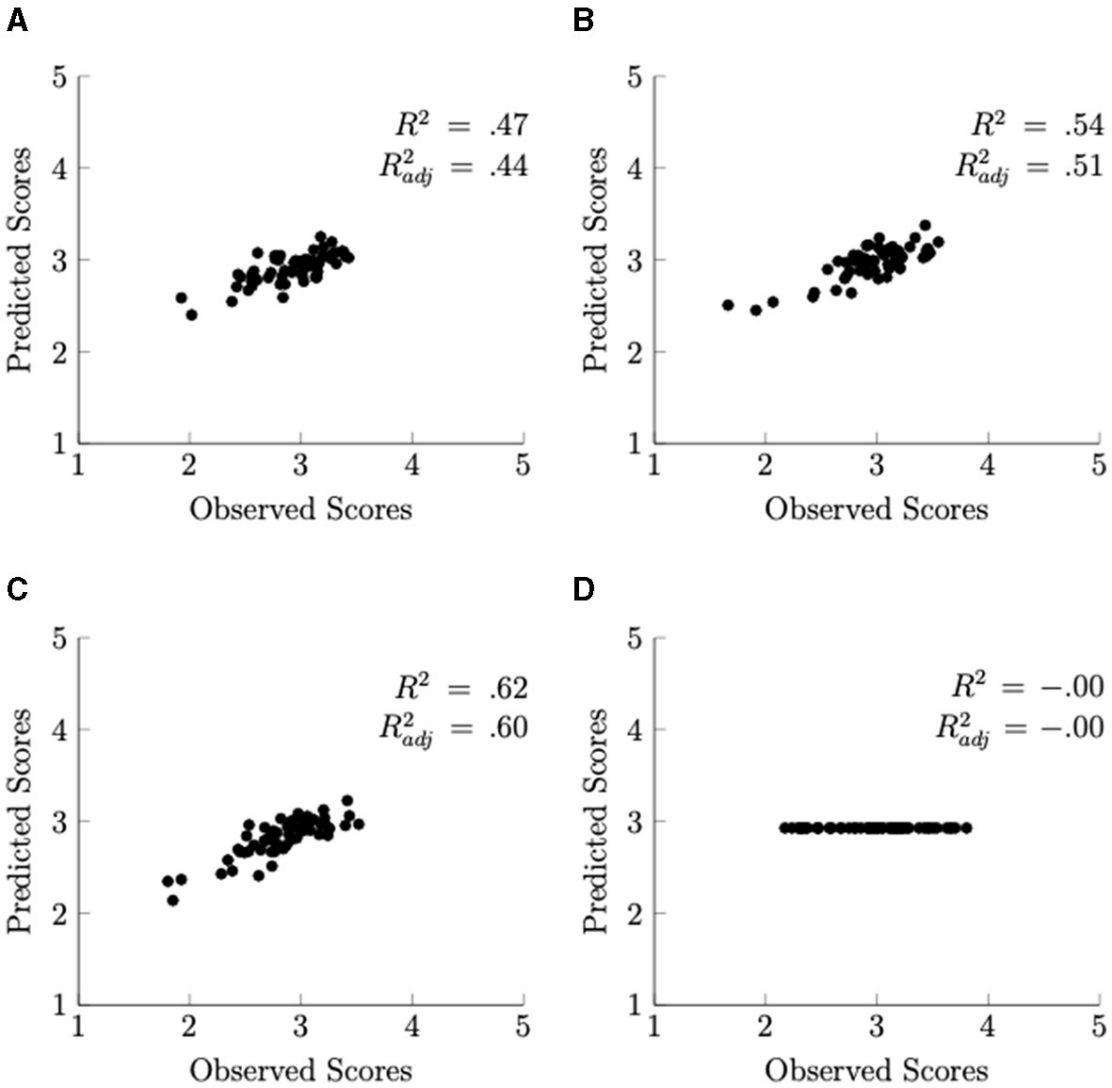
Scatterplots of the model-predicted and measured values. **(A)** Overall restorativeness. **(B)** Being away. **(C)** Fascination. **(D)** Extent.

Scatter plots of the model predicted and observed values for all 60 photographs are shown in Figure 6. For reference, the coefficient of determination (*R*^2^) and the adjusted *R*^2^ are displayed in the figure. These results indicate that the prediction accuracy was the highest for Fascination, followed by Being Away, and the Overall Restorativeness score. The Extent score was not accurately predicted by the selected model.

###  Discussion

In Study 2, we analyzed the relationship between visual properties of office photographs (such as the amount of green, color characteristics, and fractal structures) and the perceived restorativeness of the offices depicted in them. Our results showed that the amount of green, the fractal dimension of color, and the fractal dimension of brightness were significant predictors of the perceived restorativeness in office spaces. These findings are consistent with both ART and PFA, suggesting that nature-like visual properties contribute to the perception of restorativeness.

Further analysis revealed a complex relationship between the visual characteristics of office environments and their perceived restorativeness. Specifically, the amount of greenery, measured as the square root of the percentage of green area, showed a strong positive correlation with Overall Restorativeness, Being Away, and Fascination. This nonlinear relationship is consistent with previous research on density judgments (Anobile et al., 2015), suggesting that our perception of greenery in office spaces may follow similar cognitive processes.

Although the amount of greenery is a valid and visually obvious indicator of an environment's restorative properties, our lasso regression analysis revealed that the color fractal dimension is also a strong predictor of perceived restorativeness, often with a greater effect than the amount of greenery itself. In general, environments with more plants tend to have lower color fractal dimension due to the dominance of green hues and smoother color transitions, while artificial environments with abrupt color changes tend to have higher color fractal dimension. This relationship between color fractal dimension and environmental characteristics has been confirmed in previous studies (Ogawa et al., 1995; Wang and Ogawa, 2015), suggesting that color fractal dimension may serve as an indicator of the naturalness or artificiality of an environment.

The simultaneous selection of both the square root of the greenery percentage and the color fractal dimension in several models (Overall Restorativeness, Being Away, and Fascination) implies a complementary relationship between these variables. In addition, although geometric and statistical brightness fractals were selected as predictors, the magnitude of their coefficients was generally smaller than those of greenery rate and color fractal dimension. This suggests a hierarchical importance of visual properties, with the presence of greenery and overall color complexity being primary factors, and brightness complexity playing a secondary role in perceived restorativeness.

Previous studies using outdoor landscape photographs, such as Kardan et al. (2015) and Berman et al. (2014), have shown that low-level visual properties such as hue, saturation diversity, and edge density play an important role in perceptions of naturalness and environmental preference. These studies showed that images with fewer straight edges and lower saturation were perceived as more natural. Our study also supports the importance of these low-level properties, as we found a strong correlation between color fractal dimension and perceived restorativeness. Although our study did not include variables specifically measuring whether edges were straight or not, images with lower color fractal values—that is, images with less abrupt color changes—were perceived as more restorative. This finding is consistent with previous findings suggesting that a lack of straight edges contributes to the perception of naturalness and, consequently, to restorativeness.

Studies by Menzel and Reese (2021, 2022) suggest that higher-order processing, such as semantic processing of image content and concepts such as nature or urban environments, is more important than low-level visual properties for the perception of restorativeness. Our approach differs from that of Menzel and Reese (2021, 2022) in that we did not separate low-level and high-level properties in our images. Therefore, we cannot determine whether our results are due to low-level visual properties or high-level plant recognition. However, it can be interpreted that image properties such as the amount of greenery and color fractal dimensions that are not too high strongly evoke a sense of naturalness, which in turn leads to a higher sense of restorativeness.

The positive effect of natural-like environments (indicated by lower color fractal dimensions) on restorativeness is consistent with both ART and PFA. The PFA posits that the fractal nature of natural scenes contributes to perceptual fluency and asserts that such nature is restorative and preferred (Joye and van den Berg, 2011). Our results support this account, suggesting that natural environments may provide softer and more harmonious visual stimuli that promote a sense of restoration. This supports the idea that the ease of automatic attention, facilitated by certain visual properties, enhances restorativeness.

It should be noted that even in offices with high levels of greenery, scores for Overall Restorativeness, Being Away, and Fascination were only about 3.5 out of 5. This suggests that the presence of plants may be offsetting the negative ratings of non-green offices, rather than creating highly restorative environments.

Since an office is first and foremost a workplace, excessive greenery could reduce work efficiency and lead to negative evaluations. Studies by Elbertse and Steenbekkers (2023) and Larsen et al. (1998) suggest the possibility of an optimal level for indoor plants. In our study, the maximum amount of greenery in office photos was ~60%. Within this range, restorativeness scores increased as the square root of the amount of greenery increased. However, our data cannot determine whether this score would continue to increase, plateau, or decrease in offices with more than 60% greenery.

Our study did not measure actual work performance. Evensen et al. (2015) reported that while offices with plants improved perceptions of fascination, they did not positively affect performance on tasks requiring directed attention. In addition, Larsen et al. (1998) reported that while the presence of plants improved participants' ratings of the attractiveness of the office, it decreased their task performance. As their findings suggest, the presence of plants may have a negative effect on work performance. It remains unclear how restorativeness or work efficiency would be experienced when actually working in offices that were rated high on Being Away, Fascination, and Overall Restorativeness in our study. This limitation highlights the need for further research to identify the optimal level of office greenery that balances perceived restorativeness with actual work efficiency.

It is also important to note that no predictors were selected for the Extent model, suggesting that perceptions of spatial extent and coherence may be associated by factors beyond the visual properties we measured, such as actual room size or layout. This limitation highlights the need for further research on indicators corresponding to depth and extent of view.

While our study focused on visual properties measurable through photographs, we recognize the importance of other factors, such as compatibility, in office environments. Future research that integrates these aspects with our findings may provide a more comprehensive understanding of restorative office spaces.

## Practical implications

The relationship we found between visual properties and average ratings across multiple participants is particularly valuable for practical applications in office design. In a shared environment such as an office, average ratings are of considerable importance. The ability to predict these average ratings based on visual properties provides a powerful tool for designing more restorative office interiors.

The predictive model we developed based on visual properties could potentially serve as a tool for office designers and researchers, providing a method for estimating the restorative potential of an office space. The results of this study emphasize that perceived restorativeness is associated with a combination of visual properties, including the presence of greenery, color transitions, and brightness distribution, rather than a single factor. This suggests that when designing restorative office environments, attention should be paid not only to increasing greenery, but also to color complexity, such as avoiding abrupt color changes and incorporating gradual color transitions.

It should be noted that our results are based on judgments of photographs and may not fully correspond to impressions formed when actually present in an office. The narrow field of view in standard photographs is a notable difference compared to the wide range of visual information obtained when sitting at a desk. However, the practicality of photo assessments for collecting data from large samples cannot be overlooked. For future research and practical applications, it would be beneficial to develop methods for capturing photographs that more closely represent the field of view when sitting at a desk. This approach could help bridge the gap between photo-based assessments and real-life experiences, thereby increasing the applicability of this study to office design.

## Conclusion

In conclusion, our study has shown that the amount of greenery, color fractal dimension, and brightness fractal in office photographs significantly predict perceived restorativeness. The non-linear relationship between greenery and restorativeness, as well as the strong predictive power of color fractal dimension, provide new insights for office design strategies. While the results of this study provide valuable tools for estimating the restorative potential of office spaces, future research needs to address limitations such as the evaluation of the Extent component and the use of photographic assessments. This study not only contributes to the theoretical understanding of restorative environments, but also paves the way for practical applications in office design.

## Data Availability

The raw data supporting the conclusions of this article will be made available by the authors, without undue reservation.
