# Supplementary material for: Visual properties and perceived restorativeness in green offices: a photographic evaluation of office environments with various degrees of greening
Source: Front Psychol. 2024 Sep 13;15:1443540. doi: 10.3389/fpsyg.2024.1443540 (PMC11427322; doi:10.3389/fpsyg.2024.1443540)
Supplement: Supplementary file 2 [file Data_Sheet_2.pdf]

Picture Information			Number of participants	Amount of Greenery	Overall Restorativeness		Being Away		Fascination		Extent	
ID	Group	Photo (resized)	in the group	Area Percentages	Mean	SD	Mean	SD	Mean	SD	Mean	SD
7657	1	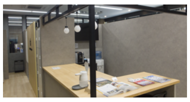	102	0.00	2.42	0.63	2.42	0.84	2.38	0.79	2.46	0.84
7543	1	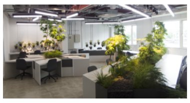	102	15.91	3.05	0.80	2.89	0.97	2.75	0.95	3.50	0.96
7602	1	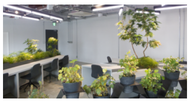	102	15.66	2.81	0.77	2.83	0.98	2.67	0.88	2.93	0.94
9664	1	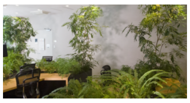	102	38.51	2.60	0.80	2.79	1.06	2.69	0.96	2.33	0.96
9664_2	1	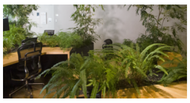	102	42.91	2.56	0.81	2.72	1.07	2.71	0.99	2.24	0.92
7673	2	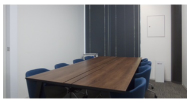	127	0.02	2.02	0.56	1.91	0.67	1.85	0.63	2.29	0.87
7664_2	2	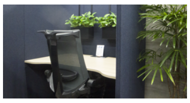	127	13.19	2.56	0.69	3.01	0.94	2.49	0.85	2.17	0.79
7558	2	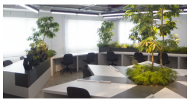	127	16.88	3.37	0.72	3.09	0.98	3.21	0.92	3.81	0.88
9645	2	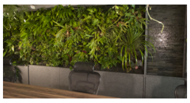	127	34.26	3.12	0.71	3.10	0.82	3.04	0.88	3.24	0.91
9663_a	2	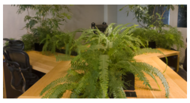	127	43.80	3.13	0.80	3.18	0.95	3.16	0.91	3.05	0.96
7666	3	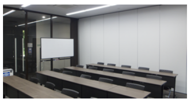	99	0.11	1.92	0.57	1.66	0.68	1.80	0.66	2.31	0.86
7621	3	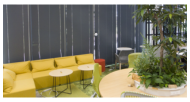	99	14.66	3.40	0.64	3.48	0.78	3.26	0.71	3.46	0.80
7636	3	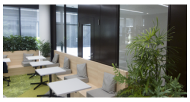	99	17.31	3.14	0.63	3.21	0.76	2.84	0.73	3.38	0.81
9646	3	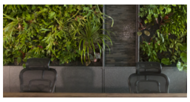	99	30.98	2.95	0.66	3.03	0.87	2.91	0.78	2.92	0.82
D1	3	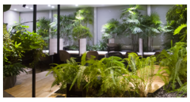	99	46.67	3.38	0.71	3.55	0.87	3.43	0.78	3.16	0.94
7644	4	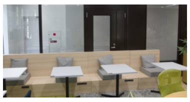	88	0.27	2.53	0.74	2.44	0.88	2.28	0.82	2.86	0.92

Picture Information			Number of participants	Amount of Greenery	Overall Restorativeness		Being Away		Fascination		Extent	
ID	Group	Photo (resized)	in the group	Area Percentages	Mean	SD	Mean	SD	Mean	SD	Mean	SD
7632	4	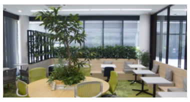	88	14.45	3.30	0.78	3.22	0.96	2.99	0.89	3.70	0.97
9648_2	4	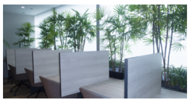	88	24.32	2.83	0.76	3.11	0.90	2.58	0.83	2.80	0.98
O1	4	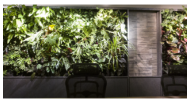	88	34.84	3.11	0.79	3.44	0.93	2.93	0.93	2.95	0.98
9662_a	4	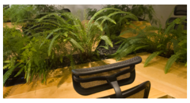	88	44.94	2.92	0.72	3.16	0.87	2.78	0.88	2.81	0.85
7648	5	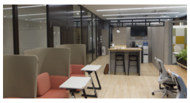	112	0.43	2.86	0.75	2.63	0.85	2.74	0.88	3.20	0.94
7674	5	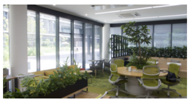	112	12.25	3.43	0.74	3.42	0.92	3.24	0.87	3.63	0.96
7567	5	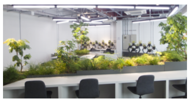	112	19.30	3.29	0.78	3.18	0.98	3.04	0.91	3.66	0.86
9652	5	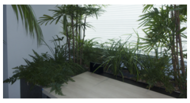	112	41.49	2.56	0.67	2.79	0.88	2.51	0.83	2.37	0.79
9644	5	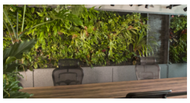	112	45.08	2.77	0.73	2.91	0.96	2.82	0.88	2.58	0.82
7551	6	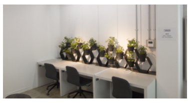	121	2.04	2.46	0.75	2.71	1.02	2.34	0.86	2.32	0.79
7645	6	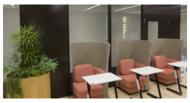	121	8.26	2.71	0.68	2.92	0.94	2.45	0.83	2.75	0.88
7640	6	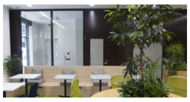	121	21.03	3.11	0.66	3.06	0.89	2.74	0.82	3.54	0.83
M1	6	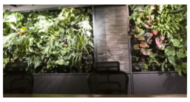	121	34.60	3.05	0.74	3.17	0.94	3.01	0.90	2.97	0.93
9666_b	6	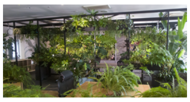	121	47.68	3.32	0.78	3.45	0.95	3.52	0.96	2.98	1.03
7670	7	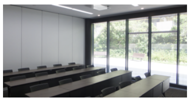	109	2.44	2.38	0.72	2.07	0.90	1.92	0.73	3.15	1.11
T7637	7	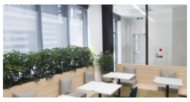	109	13.77	3.15	0.68	3.20	0.84	2.78	0.86	3.47	0.89

Picture Information			Number of participants	Amount of Greenery	Overall Restorativeness		Being Away		Fascination		Extent	
ID	Group	Photo (resized)	in the group	Area Percentages	Mean	SD	Mean	SD	Mean	SD	Mean	SD
7610	7	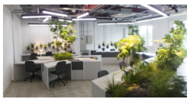	109	19.60	3.16	0.72	2.97	0.87	3.08	0.88	3.43	0.89
9651	7	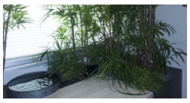	109	40.32	2.73	0.83	3.05	1.10	2.67	0.95	2.47	0.90
B3	7	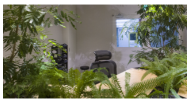	109	53.28	2.78	0.83	3.02	1.03	2.97	1.02	2.33	0.96
7603	8	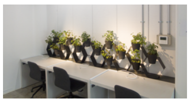	137	3.26	2.58	0.78	2.73	0.88	2.53	0.86	2.47	0.96
7574	8	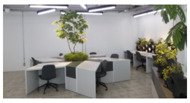	137	10.11	3.00	0.76	2.85	0.87	2.89	0.87	3.27	0.91
7562	8	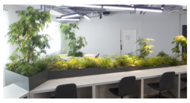	137	20.37	3.08	0.80	3.18	0.94	2.96	0.90	3.11	0.94
7611	8	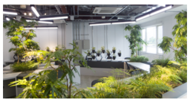	137	34.05	3.28	0.81	3.34	0.92	3.20	0.98	3.28	0.99
Q1	8	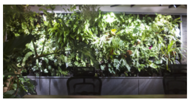	137	51.52	2.76	0.78	3.01	1.05	2.96	0.96	2.32	0.90
7633	9	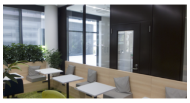	107	5.97	2.84	0.77	2.77	0.92	2.62	0.85	3.12	0.94
7649	9	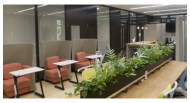	107	9.52	3.02	0.78	3.10	0.99	2.73	0.86	3.22	0.99
9653	9	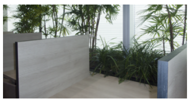	107	25.76	2.61	0.73	2.93	0.91	2.53	0.86	2.37	0.82
R1	9	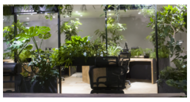	107	32.65	3.28	0.83	3.46	0.93	3.21	0.95	3.17	0.91
9667	9	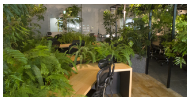	107	51.97	3.02	0.87	3.19	1.08	3.18	1.06	2.67	0.98
7547	10	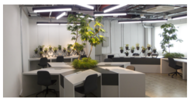	98	6.07	2.94	0.77	2.84	0.90	2.91	0.93	3.05	1.00
7590	10	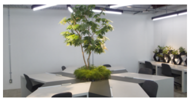	98	9.61	2.80	0.74	2.65	0.89	2.74	0.90	3.01	0.96
7619	10	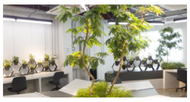	98	22.13	3.20	0.77	3.30	0.90	3.11	0.94	3.20	0.97

Picture Information			Number of participants	Amount of Greenery	Overall Restorativeness		Being Away		Fascination		Extent	
ID	Group	Photo (resized)	in the group	Area Percentages	Mean	SD	Mean	SD	Mean	SD	Mean	SD
P1	10	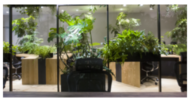	98	28.67	3.26	0.78	3.46	0.93	3.40	0.86	2.92	0.99
A1	10	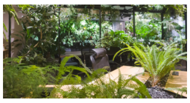	98	60.18	3.18	0.85	3.43	1.03	3.42	1.00	2.68	1.03
7583	11	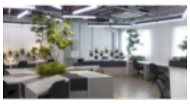	115	8.22	3.19	0.78	2.91	0.96	3.01	0.92	3.67	0.97
7641_a	11	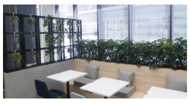	115	16.21	3.03	0.71	3.09	0.83	2.76	0.85	3.22	0.89
7582	11	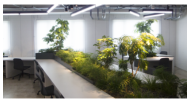	115	25.63	3.00	0.82	2.97	0.97	2.88	0.99	3.15	0.94
9655	11	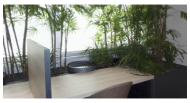	115	29.55	2.81	0.83	3.11	0.96	2.73	0.91	2.59	0.94
9666_2	11	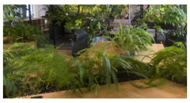	115	59.92	2.92	1.01	3.10	1.25	3.07	1.12	2.57	1.12
7628	12	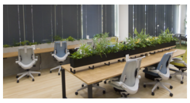	107	7.19	2.85	0.70	2.75	0.85	2.64	0.81	3.17	0.91
7660	12	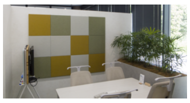	107	7.55	2.43	0.70	2.56	0.80	2.43	0.82	2.31	0.81
7570	12	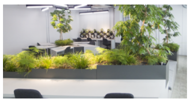	107	25.34	3.11	0.77	3.14	0.93	3.06	0.84	3.15	0.94
L1	12	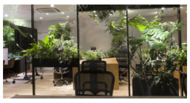	107	31.24	3.16	0.85	3.22	1.00	3.25	0.93	3.01	1.05
7563	12	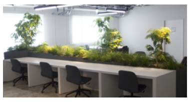	107	13.98	3.01	0.71	2.98	0.86	2.87	0.79	3.18	0.88
