# Supplementary material for: Visual properties and perceived restorativeness in green offices: a photographic evaluation of office environments with various degrees of greening
Source: Front Psychol. 2024 Sep 13;15:1443540. doi: 10.3389/fpsyg.2024.1443540 (PMC11427322; doi:10.3389/fpsyg.2024.1443540)
Supplement: Supplementary file 4 [file Data_Sheet_4.docx]

### Square Root of Greenery

The square root of the percentage of green pixels relative to the total number of pixels in the image.

### Mean Brightness

Mean brightness value of all pixels in the image. The image was pre-converted to HSV color space, and brightness was calculated using the converted image. Higher values indicate a brighter overall image, while lower values suggest a darker image.

### Mean Saturation

Mean saturation value of all pixels in the image. The image was pre-converted to HSV color space, and saturation was calculated using the converted image. Higher values indicate more vivid and intense colors, while lower values suggest more muted or grayish colors.

### Shape Fractal

Shape fractal measures the complexity and self-similarity of the image's edges and shapes. After applying the Edge Glow filter in Adobe Photoshop, the image was converted to the YCrCb color space and then transformed into an 8-bit grayscale image using the luminance value of the converted image. The grayscale image was then binarized by setting pixels with gray levels at or below the average to black and those above the average to white. Using the binarized black and white image, the fractal dimension was calculated using the box-counting method, counting the number of white pixels in grid sizes from 1 to saturation, following the method of Wu et al. (2020). Higher shape fractal values indicate more complex and intricate edge patterns, while lower values suggest simpler, more uniform shapes.

### Geometric Fractal, Cubic Method

Geometric fractal dimension quantifies the spatial complexity of the image using a three-dimensional approach. The image was converted to the HSV color space and then to an 8-bit grayscale image based on either brightness (value) or saturation. The image was then subjected to the cube method (Nakayama et al., 1989).

The geometric fractal dimension was calculated separately for box sizes from 1 to 70 pixels (small), for larger sizes (large), and overall (1 to 256 pixels). This approach allows for a multi-scale analysis of the image, capturing both fine local features (1-70 pixels) and global structures (>70 pixels). Higher geometric fractal values indicate more complex and detailed spatial structures at various scales, while lower values suggest more uniform or simple spatial patterns.

### Statistical Fractal, Brownian Motion Method

Statistical fractal dimension measures the roughness and irregularity of the image using a statistical approach. The image was converted to the HSV color space and then to an 8-bit grayscale image based on either brightness (value) or saturation. The image was then subjected to the Brownian Motion Method (Stewart et al., 1993).

The statistical fractal dimension was calculated separately for box sizes from 1 to 300 pixels (small), for larger sizes (large), and overall (1 to 2048 pixels). This approach allows for a multi-scale analysis of the image, capturing both fine local features (1-300 pixels) and global structures (>300 pixels). Higher statistical fractal values indicate more irregular and rough textures across different scales, while lower values suggest smoother and more regular patterns.

### Color Fractal, Power Spectral Analysis

Color fractal dimension quantifies the complexity of color distribution in the image. For the power spectrum, the magnitude of the position vector of each pixel in the CIE color space was taken as the pixel value. The fractal dimension was calculated from the slope of the log-log plot of the spectral density obtained by performing a two-dimensional discrete cosine transform and the spatial composite frequency. Higher color fractal values indicate more complex and varied color distributions, while lower values suggest more uniform or simple color patterns.

### 1/f Fluctuation

1/f fluctuation measures the balance between randomness and order in the image's grayscale values. The 8-bit grayscale-converted image was subjected to a two-dimensional Fourier transform to obtain a log-log plot of the power spectrum values and frequencies. The absolute value of the slope of the regression line was taken as the fluctuation value α (Izumi et al., 2010). The standard deviation of the residuals was also used as a parameter. These values were measured across multiple frequency ranges. A fluctuation value α close to 1 indicates a balance between randomness and order, often associated with natural and aesthetically pleasing images. Values significantly higher or lower than 1 suggest either too much randomness or too much order, respectively.

**References**

Izumi T., Hattori T., Fujita E., Sugimoto S. & Kawano H., (2010). Feeling impression and quantities accompanying calculation of fluctuation in color image. *Journal of Japan Society of Kansei Engineering, 9*, 2, 243–250.

Nakayama H., Sone M., & Takagi M., (1989). Analysis and Evaluation of Meteorological Satellite NOAA Image by Fractal Dimension and Lower Order Statistics. *Journal of Information Processing Society of Japan, 30*, 1, 91–100.

Stewart C. V., Moghaddam B., Hintz K. J. & Novak L. M., (1993). Fractional Brownian motion models for synthetic aperture radar imagery scene segmentation. *Proceedings of IEEE, 81*, 10, 1511–1522, 1993

Wu J., Jin X., Mi S., & Tang J., (2020). An effective method to compute the box-counting dimension based on the mathematical definition and intervals. *Result in Engineering, 6*, 100106.
